# Case Report: The revelation of a new pathogenic variant in the POT1 gene in a patient with a pediatric high-grade glioma and a renal cell carcinoma

**DOI:** 10.3389/fonc.2026.1778785

**Published:** 2026-05-18

**Authors:** Selene Cipri, Antonella Cacchione, Annalisa Serra, Giada Del Baldo, Evelina Miele, Lucia Pedace, Sara Patrizi, Federica D’Antonio, Emanuele Agolini, Antonio Novelli, Alessandro Crocoli, Arianna Bertocchini, Andrea Carai, Sabina Barresi, Sabrina Rossi, Rita Alaggio, Francesca Diomedi Camassei, Giovanna Stefania Colafati, Luigi Boccuto, Angela Mastronuzzi

**Affiliations:** 1 Hematology/Oncology, Cell Therapy, Gene Therapies and Hemopoietic Transplant Area, Bambino Gesù Pediatric Hospital, Istituto di Ricovero e Cura a Carattere Scientifico (IRCCS), Rome, Italy; 2 Laboratory of Medical Genetics Area, Bambino Gesù Pediatric Hospital, Istituto di Ricovero e Cura a Carattere Scientifico (IRCCS), Rome, Italy; 3 Emergency General Surgery, Bambino Gesù Pediatric Hospital, Istituto di Ricovero e Cura a Carattere Scientifico (IRCCS), Rome, Italy; 4 Neurosurgery Area, Bambino Gesù Pediatric Hospital, Istituto di Ricovero e Cura a Carattere Scientifico (IRCCS), Rome, Italy; 5 Pathological Anatomy Area, Bambino Gesù Pediatric Hospital, Istituto di Ricovero e Cura a Carattere Scientifico (IRCCS), Rome, Italy; 6 Nuclear Medicine Area, Bambino Gesù Pediatric Hospital, Istituto di Ricovero e Cura a Carattere Scientifico (IRCCS), Rome, Italy; 7 School of Nursing, College of Behavioral, Social and Health Sciences, Clemson University, Clemson, SC, United States; 8 Department of Life Sciences and Public Health, Catholic University of the Sacred Heart, Rome, Italy

**Keywords:** brain tumors, cancer predisposition syndrome, high-grade glioma, kidney cancer, POT1

## Abstract

This study aimed to describe and molecularly characterize a rare case of early-onset multiple primary tumors associated with a novel germline *protection of telomeres 1* (*POT1*) gene pathogenic variant, associated with POT1 tumor predisposition syndrome (POT1-TPD), a rare autosomal dominant disorder characterized by an increased risk for various tumors, including gliomas. A custom clinical exome panel for genes associated with Cancer Predisposition Syndromes was performed on DNA extracted from blood. RNA sequencing and genome-wide DNA methylation profiling were conducted on tumors. We report the case of a female patient diagnosed at age 12 with a diffuse glioma harboring a ROS1 fusion. At age 18, she developed a renal cell carcinoma. Genetic germline testing revealed a heterozygous germline *POT1* variant, c.910dupG (p.Asp304fs*8), classified as pathogenic. Segregation analysis demonstrated paternal inheritance of the variant. The same POT1 variant was identified in both tumor tissues in a heterozygous state. This is the first reported case of a young adult carrying a new pathogenic *POT1* variant who developed a pediatric high-grade glioma and a renal tumor in early adulthood. This report expands the clinical spectrum of POT1-associated tumors with early-onset and underscores the relevance of genetic testing for patient management and family counseling.

## 1 Introduction

POT1 tumor predisposition syndrome (POT1-TPD, OMIM #615848) is a rare syndrome with autosomal dominant transmission, associated with pathogenic variants in the *POT1* (*protection of telomeres 1*) gene, with unknown penetrance and a lack of established genotype-phenotype correlation (1). To date, the prevalence of this syndrome is unknown, and about a hundred familial cases are described in the literature (1). Germinal alterations are associated with several cancers, such as glioma (2–4). The nuclear protein encoded by the *POT1* gene is a member of the telomeric shelterin multiprotein complex that, through binding to the 5′-TTAGGG-3′ tandem repeats of telomeres, has the function of protecting them (5). Telomeres play a key role in maintaining genome stability in physiological conditions, while most cancer cells exploit such a mechanism, known as TMM (telomere maintenance mechanism), to avoid over-shortening of telomeres during their high-speed proliferation (5).

Therefore, in addition to a carcinogenic predisposition encompassing a wide range of tumor types, POT1-TPD upholds remarkable clinical and research value as an intriguing disease model to deepen our understanding of one of the most efficacious and deleterious mechanisms underlying cancer proliferation.

Here, we described the first case of a young adult with a POT1-TPD who developed a diffuse glioma with *ROS1* fusion and later a renal cell carcinoma (RCC).

## 2 Case presentation

We present a case of a Caucasian girl who developed two distinct primary tumors: a brain tumor in childhood and a renal tumor in adolescence. At age 12, she presented with drowsiness and vomiting. Brain MRI revealed a mass, and histopathological examination led to a diagnosis of glioneuronal tumor with focal anaplasia, not otherwise specified (NOS) (see **Supplementary Figure 1**). The patient underwent radiotherapy combined with temozolomide treatment.

At age 18, during routine magnetic resonance imaging (MRI) follow-up for the brain tumor, imaging revealed a left renal mass, which was confirmed as RCC, Grade 2 according to WHO/ISUP and TNM T1b, N0, Mx. Molecular testing did not reveal any fusions or other alterations using the Archer FusionPlex panel (see **Supplementary Figure 2**). The patient subsequently underwent nephrectomy.

At the time of this report, she remains in good health and is not receiving any treatment, 7 years post-brain tumor diagnosis and 1 year post-renal tumor diagnosis.

**Figure 1:**
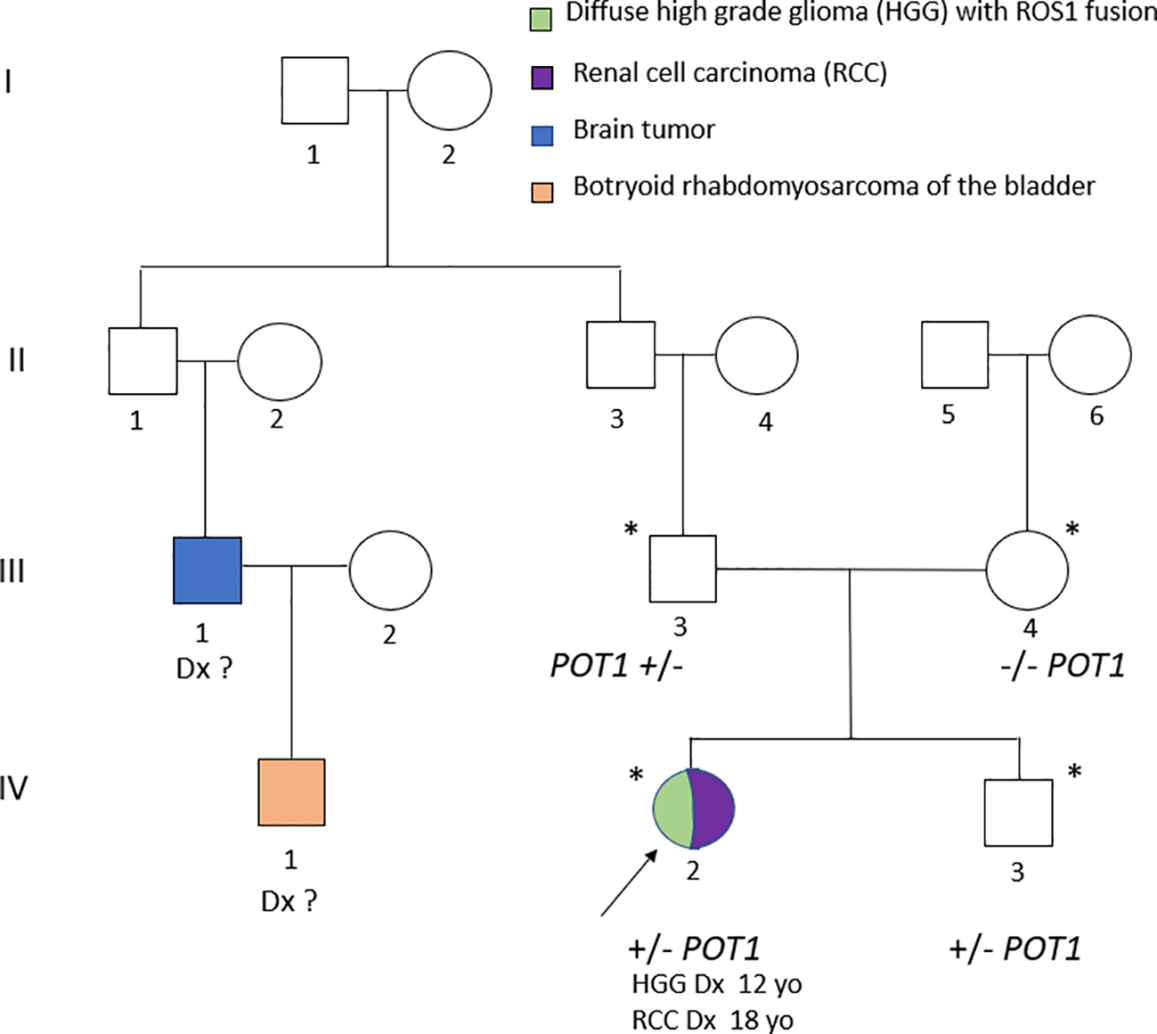
Pedigree of the family. An asterisk indicates the individuals who received DNA sequencing. A plus (+) sign indicates the detection of the NM_015450.2 (*POT1*): c.910dupG (p.Asp304fs*8).

Family history was notable for the father’s cousin diagnosed with a brain tumor in infancy, and one second-degree paternal relative affected by a botryoid rhabdomyosarcoma of the bladder healed. The patient’s parents and younger brother are currently in good health (**Figure 1**).

**Figure 2:**
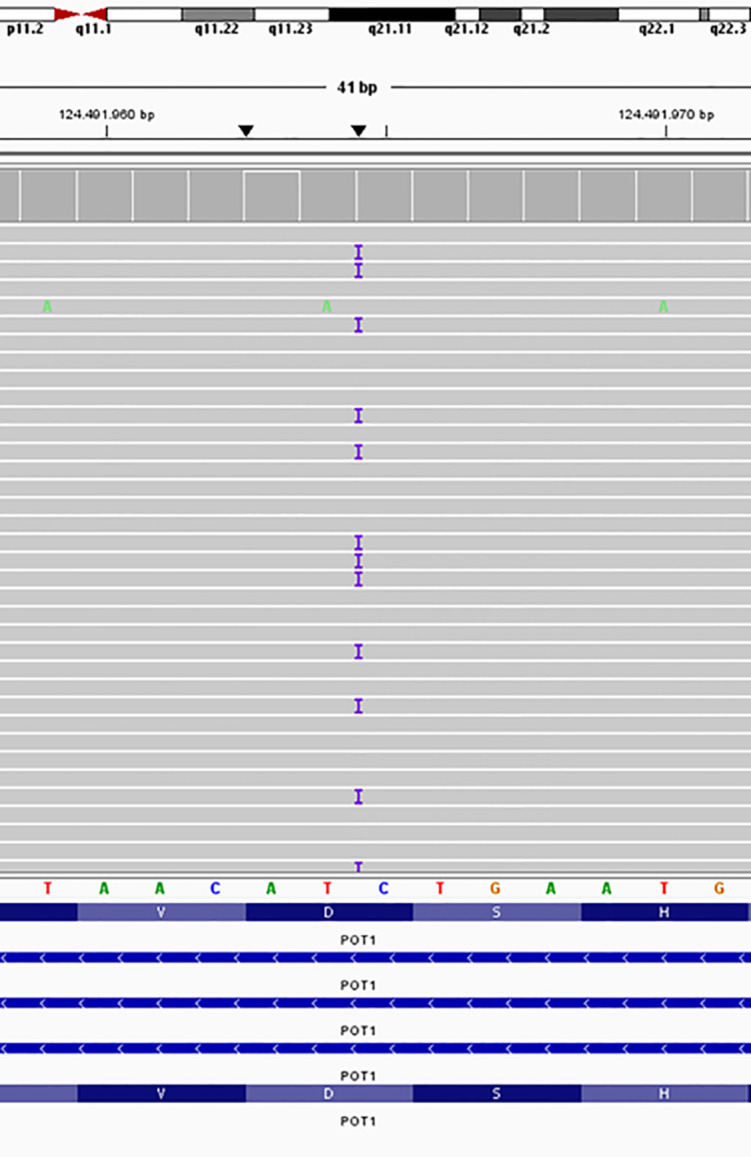
IGV screenshot of exome sequencing alignment showing the variant NM_015450.2 (*POT1*): c.910dupG (p.Asp304fs*8).

Genetic testing using a clinical exome panel for cancer predisposition syndromes (see **supplementary material and methods**) identified a heterozygous pathogenic variant in the *POT1* gene (OMIM:*606478): c.910dupG (p.Asp304fs*8), based on transcript NM_015450.2 (**Figure 2**). The variant inserts a guanine nucleotide at position 910, causing a frameshift that introduces a premature stop codon 8 amino acids downstream of the mutation. This variant was inherited from the patient’s father and is absent from ClinVar, gnomAD, and the published literature. The *POT1* variant was detected in both the brain and renal tumor tissues without loss of heterozygosity (LOH). Deleterious germline *POT1* variants are associated with POT1-TPD, which confers increased lifetime risk of gliomas, multiple cutaneous melanomas, chronic lymphocytic leukemia, and angiosarcoma (1, 2). Given these findings, the father and brother - although asymptomatic - underwent comprehensive clinical screening (prostate, kidney, thyroid, bladder, and colon evaluations), all of which were unremarkable to date.

Additionally, a heterozygous variant of uncertain significance (VUS) in the *DICER1* gene - c.3713T>C (p.Leu1238Pro), maternally inherited - was also identified in the patient’s germline and found in the brain tumor tissue (allele frequency: 47%).

Molecular analysis of the brain tumor (see **supplementary material and methods**) revealed several clinically relevant copy number alterations (CNAs), including gains involving *CDK4*, *CDK6*, and *FGF14* (four copies each). Tumor mutational burden (TMB) was low at 1.6 mutations/Mb, and microsatellite instability (MSI) was 2%. Fusion analysis using targeted RNA sequencing identified a translocation between *GOPC* (exon 8) and *ROS1* (exon 35) [11 reads*].

**Figure 3:**
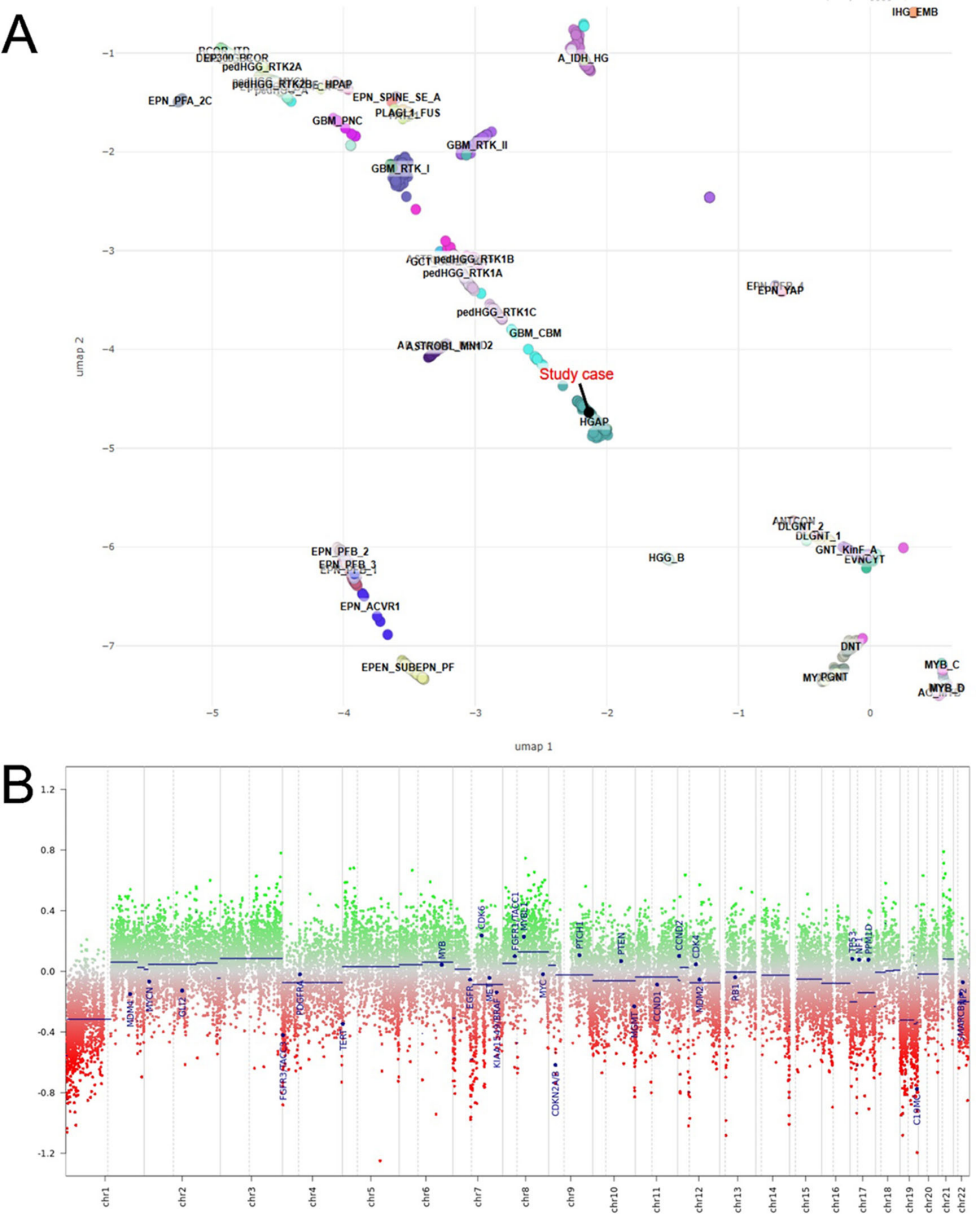
DNA methylation profiling of the study case. **(A)** UMAP plot generated through the Bethesda classifier, zoomed in to show that the study sample clusters with HGAP. **(B)** CNV plot generated from DNA methylation data. Chromosomes 1–22 are represented on the X axis, with the centromeres represented by a dotted line. Positive deviations from 0 (green) represent gains/amplifications, while negative deviations represent losses.

DNA methylation profiling (see **supplementary material and methods**) revealed no match to any defined class using the DKFZ/Heidelberg Brain Tumor Classifier v12.8. However, the Bethesda Central Nervous System (CNS) Tumor Classifier v2.0 categorized the tumor as high-grade glioma with piloid features (HGAP) with high confidence (score: 0.95; UMAP Bethesda, **Figure 3A**). CNA analysis inferred from the methylation array showed losses of chromosomes 1p, 4, 7, 19, and 22, and a focal deletion at the *CDKN2A/B* locus (**Figure 3**). The Heidelberg classifier identifies brain tumor as an HGAP, but with a very low score (0.37). Based on integrated histopathological, molecular, and epigenetic data, the final diagnosis for the brain tumor was diffuse glioma with *ROS1* fusion, not elsewhere classified (NEC) (6). Finally, the initial diagnosis at age 12 was based on histopathological evaluation with limited molecular data. Following advanced molecular profiling (RNA sequencing and DNA methylation analysis), the tumor was reclassified as diffuse glioma with *ROS1* fusion, NEC according to integrated WHO criteria. Timeline of clinical presentation, diagnostic assessment, therapeutic interventions, follow-up, and outcomes according to CARE guidelines is reported in **Table 1**.

**Table 1:** Timeline of diagnostic evaluation, therapeutic interventions, follow-up, and outcomes.

Age	Timepoint	Clinical event
12 years	Clinical presentation	Onset of drowsiness and vomiting
12 years	Diagnostic imaging	Brain MRI revealed an intracranial mass
12 years	Histopathological diagnosis	Glioneuronal tumor with focal anaplasia, not otherwise specified (NOS)
12 years	Molecular analysis (tumor)	GOPC–ROS1 fusion identified; low tumor mutational burden (1.6 mutations/Mb); microsatellite stability (MSI 2%); copy number gains involving CDK4, CDK6, and FGF14
12 years	DNA methylation profiling	Classified as high-grade glioma with piloid features (HGAP) by Bethesda CNS Tumor Classifier (score 0.95); no definitive class assignment by Heidelberg classifier
12 years	Integrated diagnosis	Diffuse glioma with ROS1 fusion, not elsewhere classified (NEC)
12 years	Therapeutic intervention	Radiotherapy combined with temozolomide
12–18 years	Follow-up	Regular brain MRI follow-up with no evidence of disease progression
18 years	Incidental finding	Routine MRI follow-up revealed a left renal mass
18 years	Diagnostic assessment	Imaging and histopathology confirmed renal cell carcinoma
18 years	Therapeutic intervention	Left nephrectomy
18 years	Germline genetic testing	Heterozygous pathogenic variant in *POT1*: c.910dupG (p.Asp304fs*8), inherited from father
18 years	Tumor genetic findings	*POT1* variant detected in both brain and renal tumor tissues without loss of heterozygosity
18 years	Additional genetic findings	Germline *DICER1* variant of uncertain significance (c.3713T>C; p.Leu1238Pro), maternally inherited
19 years	Family evaluation	Father and brother underwent clinical screening; no abnormalities detected
19 years	Outcome	Patient in good health and off therapy, 7 years after brain tumor diagnosis and 1 year after renal tumor diagnosis

## 3 Discussion

To our knowledge, this is the first case of a young adult reported in the literature with a pathogenic variant in the *POT1* gene who developed a diffuse glioma with *ROS1* fusion at pediatric age and a renal tumor in the young adult stage. Family history reported botryoid rhabdomyosarcoma of the bladder and a pediatric brain tumor in one second-degree relative on the paternal side and in a cousin of the father, respectively. Moreover, the latter were not subjected to genetic testing and/or search for the familial pathogenic mutation found in the *POT1* gene. Three unrelated cases were reported in the literature, in which at least two family members of each case had a brain tumor. In two of these three cases, other family members developed different types of cancer, including kidney cancer (2). In addition, it has recently been described the first case of an adult patient with RCC, glioma, and colon polyps, carrying a pathogenic *POT1* variant that has not been previously reported in the literature nor in population databases, just like the variant detected in our patient (7). Public datasets such as TCGA and cBioPortal include very limited information on germline *POT1* variants, precluding robust comparative analyzes. This highlights the need for dedicated registries. On the other hand, a large-scale study on 19,315 patients with cancer from the Oncology Research Information Exchange Network (ORIEN) has been conducted (8). This study confirmed the association between germline *POT1* variants and an increased risk of CLL and papillary thyroid carcinoma, with diagnosis occurring at a younger age (8). The use of an unselected pan-cancer cohort is noteworthy, as it reduces selection bias and underscores the need for further studies in unselected populations.

Finally, *POT1* is expressed in the human embryonic kidney, and some studies have shown upregulation of *POT1* mRNA in renal cell cancer (7, 9). In addition, a correlation between *POT1* dysregulation and cancer telomere pathology is relevant in several types of tumors (3, 4). The Telomeres Mendelian Randomization Collaboration also analyzed variants in the *POT1* gene associated with telomere length, showing a causative impact between longer telomeres and the risk of cancers, including glioma and renal cancer (10, 11). Telomere length analysis was not performed in this patient. This represents a limitation, as POT1 pathogenic variants are typically associated with telomere elongation. Consequently, we cannot exclude that renal cancer, developed secondary to a high-grade glioma tumor, is associated with the diagnosis of POT1-TDP and thus linked to the pathogenic *POT1* variant.

In conclusion, POT1 tumor predisposition syndrome is a rare condition; therefore, further studies are needed to define the risk of developing second cancers with an early onset, such as renal cell carcinoma, in patients diagnosed with a diffuse glioma with *ROS1* fusion. A better understanding of risk would improve follow-up strategies in patients and family members sharing *POT1* variants.

## Data Availability

The datasets presented in this study can be found in online repositories. The names of the repository/repositories and accession number(s) can be found in the article/**Supplementary Material**.
