# Supplementary material for: Case Report: The revelation of a new pathogenic variant in the POT1 gene in a patient with a pediatric high-grade glioma and a renal cell carcinoma
Source: Front Oncol. 2026 May 18;16:1778785. doi: 10.3389/fonc.2026.1778785 (PMC13222780; doi:10.3389/fonc.2026.1778785)
Supplement: Supplementary file 1 [file DataSheet1.docx]

Supplementary Material

# Supplementary Materials and methods

***Germinal testing and data analysis***

Genomic DNA was extracted from peripheral blood (collected from the patient’s parents) samples by using “DNA blood mini kit” (Qiagen, Hilden, NW, Germany), according to the manufacturer’s instructions. The quantification of DNA was performed by using Qubit fluorimeter (Life Technologies, Carlsbad, California, USA) with the dsDNA HS Assay Kit, following the manufacturer’s instructions. A custom clinical exome panel for the genes associated with the Cancer Predisposition Syndromes (Twist Bioscience, South San Francisco, CA, USA) was performed through Next Generation Sequencing by using NovaSeq 6000 platform (Illumina, San Diego, CA, USA). The genes analyzed are: *FGFR1* (NM_023110.3), *FGFR3* (NM_000142.5), *LZTR1* (NM_006767.4), *MGMT* (NM_002412.5), *NFKBIA* (NM_020529.3), *PPARG* (NM_138711.6), *TACC3* (NM_006342.3), *TP53* (NM_000546.5), *IDH1* (NM_005896.4), *ERBB2* (NM_004448.4), *PTEN* (NM_000314), *EGFR* (NM_005228), *NF1* (NM_000267.3), *NF2* (NM_000268), *POT1* (NM_015450), *BRCA2* (NM_000059), *MLH1* (NM_000249), *MSH2* (NM_000251), *EPCAM* (NM_002354), *PMS2* (NM_000535), *MSH6* (NM_000179), *MLH3* (NM_001040108), *CDK4* (NM_000075), *PDGFRA* (NM_006206), *PIK3CA* (NM_006218), *ALK* (NM_004304.4), *ACVR1* (NM_001105), *BRAF* (NM_004333), *VHL* (NM_000551), *KIF1B* (NM_015074), *TMEM127* (NM_001193304), *RET* (NM_020975), *MAX* (NM_002382), *SDHA* (NM_004168), *SDHB* (NM_003000), *SDHC* (NM_003001), *SDHAF2* (NM_017841), *SDHD* (NM_003002), *SLC5A11* (NM_052944), *MDM2* (NM_002392), *CASP10* (NM_001230.4), *DICER1* (NM_030621.4), *APC* (NM_000038.6), *ATM* (NM_000051.3), *BRCA1* (NM_007294.4), *CDH1* (NM_004360.5), *CHEK2* (NM_007194.4), *MSH3* (NM_002439.5), *MUTYH* (NM_012222.2), *NBN* (NM_002485.5), *PALB2* (NM_024675.4), *POLE* (NM_006231.4), *TSC1* (NM_000368.5), *TSC2* (NM_000548.4).

***NGS analysis in tumor tissue***

***RNA Sequencing analysis***

RNA was extracted using the ReliaPrep FFPE total RNA kit (Promega, Madison, USA), according to the manufacturer’s protocol, starting from FFPE sections containing at least 80% of neoplastic cells. RNA concentrations were measured on a Qubit 2.0 Fluorometer (Thermofisher Scientific, Waltham, USA) using the Qubit dsDNA High Sensitivity. A comprehensive profiling assay was performed in Next Generation Sequencing analysis and targeting 52 fusion genes. NGS data were analyzed with Archer FusionPlex OPBG custom panel, Illumina platform (Miseq). Data analysis was obtained using Archer® Analysis 6.0 software.

***DNA Sequencing analysis***

DNA was extracted from formalin-fixed paraffin-embedded (FFPE) tumor tissue using NucleoSpin Tissue Kit (Machery‑Nagel) according to the manufacturer’s protocol; DNA concentrations were measured using a Qubit 2.0 Fluorometer (Thermo Scientific), and dsDNA High Sensitivity assay. DNA libraries were prepared according to the TSO500 Library Preparation Kit (Illumina, San Diego, CA, USA). DNA libraries were pooled in equimolar amounts of 4 nM (8 libraries/pool), denatured, appropriately diluted, loaded in a paired-end mode (2 X 101-bp reads) using NEXTSEQ 550 platform (Illumina, San Diego, California), and sequenced to a mean coverage depth of >500× for up to 500 cancer-related genes. NGS data were analyzed with Illumina TruSight Oncology 500 Local App v2.1, and variant report files were uploaded into the Pierian Clinical Genomics Workspace cloud (Pierian DX software CGW_V6.21.1).

***Genome-wide DNA methylation profiling analysis***

DNA methylation profiling was performed on the brain tumor sample using DNA extracted from formalin-fixed, paraffin-embedded (FFPE) tissue, following established protocols (1). A total of 250 ng of DNA was used as input for analysis with the Infinium MethylationEPIC BeadChip v1.0 (850K array; Illumina), according to the manufacturer’s instructions. Raw methylation intensity data files (IDATs) were uploaded to versions 12.5 and 12.8 of the DKFZ/Heidelberg Brain Tumor Classifier platform (2,3), as well as to the preliminary "Bethesda CNS tumor classifier v2.0" for additional classification insights. Copy number alteration (CNA) analysis was conducted using the Conumee R package (4), following previously described methodologies (5).

# Supplementary Figures and Tables


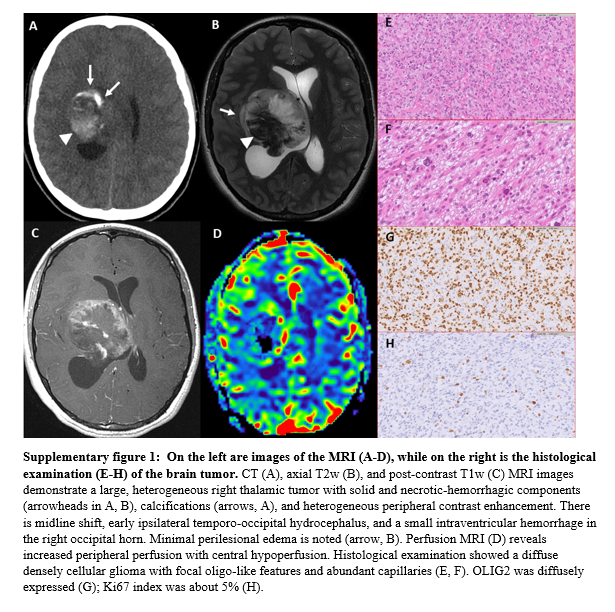


**Supplementary figure 1: On the left are images of the MRI (A-D), while on the right is the histological examination (E-H) of the brain tumor.** CT (A), axial T2w (B), and post-contrast T1w (C) MRI images demonstrate a large, heterogeneous right thalamic tumor with solid and necrotic-hemorrhagic components (arrowheads in A, B), calcifications (arrows, A), and heterogeneous peripheral contrast enhancement. There is midline shift, early ipsilateral temporo-occipital hydrocephalus, and a small intraventricular hemorrhage in the right occipital horn. Minimal perilesional edema is noted (arrow, B). Perfusion MRI (D) reveals increased peripheral perfusion with central hypoperfusion. Histological examination showed a diffuse densely cellular glioma with focal oligo-like features and abundant capillaries (E, F). OLIG2 was diffusely expressed (G); Ki67 index was about 5% (H).


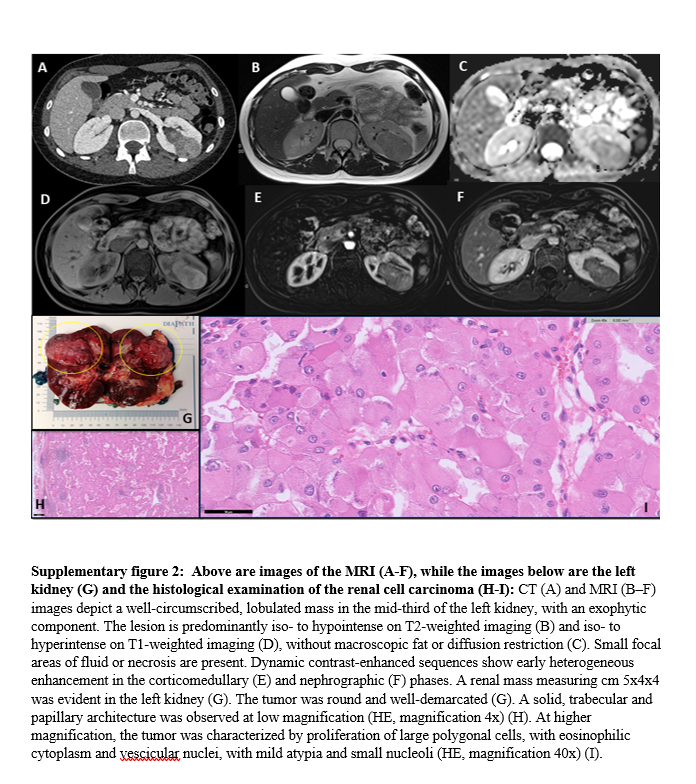


**Supplementary figure 2: Above are images of the MRI (A-F), while the images below are the left kidney (G) and the histological examination of the renal cell carcinoma (H-I):** CT (A) and MRI (B–F) images depict a well-circumscribed, lobulated mass in the mid-third of the left kidney, with an exophytic component. The lesion is predominantly iso- to hypointense on T2-weighted imaging (B) and iso- to hyperintense on T1-weighted imaging (D), without macroscopic fat or diffusion restriction (C). Small focal areas of fluid or necrosis are present. Dynamic contrast-enhanced sequences show early heterogeneous enhancement in the corticomedullary (E) and nephrographic (F) phases. A renal mass measuring cm 5x4x4 was evident in the left kidney (G). The tumor was round and well-demarcated (G). A solid, trabecular and papillary architecture was observed at low magnification (HE, magnification 4x) (H). At higher magnification, the tumor was characterized by proliferation of large polygonal cells, with eosinophilic cytoplasm and vescicular nuclei, with mild atypia and small nucleoli (HE, magnification 40x) (I).

**References**

1. Pedace L, Pizzi S, Abballe L, Vinci M, Antonacci C, Patrizi S, et al. Evaluating cell culture reliability in pediatric brain tumor primary cells through DNA methylation profiling. Npj Precis Oncol. 2024 Apr 18;8(1):92.

2. molecularneuropathology.org/mnp/ [Internet]. [cited 2025 June 5]. Available from: https://www.molecularneuropathology.org/mnp/

3. Capper D, Jones DTW, Sill M, Hovestadt V, Schrimpf D, Sturm D, et al. DNA methylation-based classification of central nervous system tumours. Nature. 2018 Mar 22;555(7697):469–74.

4. The conumee vignette [Internet]. [cited 2025 June 5]. Available from: https://bioconductor.org/packages/devel/bioc/vignettes/conumee/inst/doc/conumee.html

5. Antonacci C, Abballe L, Patrizi S, Pedace L, Barresi S, Giovannoni I, et al. DNA methylation profiling from cerebrospinal fluid as a diagnostic tool for pineoblastoma. Acta Neuropathol Commun. 2025 Mar 8;13(1):52.
